# Risk of neurologic events after surgery for mitral valve insufficiency and concomitant Cox-maze IV procedure for atrial fibrillation. A nationwide register-based study

**DOI:** 10.1093/icvts/ivae189

**Published:** 2024-11-18

**Authors:** Anders Albåge, Farkas Vanky, Gabriella Boano, Anders Holmgren, Lena Jidéus, Birgitta Johansson, Göran Kennebäck, Shahab Nozohoor, Henrik Scherstén, Johan Sjögren, Anders Wickbom, Torbjörn Ivert

**Affiliations:** Department of Cardiothoracic Surgery and Anesthesiology, University Hospital, Uppsala, Sweden; Department of Surgical Sciences, Uppsala University, Uppsala, Sweden; Department of Thoracic and Vascular Surgery, Linköping University, Linköping, Sweden; Unit of Cardiovascular Medicine, Department of Health, Medicine and Caring Sciences, Linköping University, Linköping, Sweden; Department of Thoracic and Vascular Surgery, Linköping University, Linköping, Sweden; Unit of Cardiovascular Medicine, Department of Health, Medicine and Caring Sciences, Linköping University, Linköping, Sweden; Heart Centre and Department of Public Health and Clinical Medicine, Umeå University, Umeå, Sweden; Department of Cardiothoracic Surgery and Anesthesiology, University Hospital, Uppsala, Sweden; Department of Surgical Sciences, Uppsala University, Uppsala, Sweden; Department of Medicine, Geriatrics and Emergency Medicine, Sahlgrenska University Hospital/Östra, Gothenburg, Sweden; Department of Cardiology, Karolinska University Hospital, Karolinska Institutet, Stockholm, Sweden; Department of Clinical Sciences Lund, Skåne University Hospital, Lund University, Lund, Sweden; Department of Cardiothoracic Surgery, Skåne University Hospital, Lund University, Lund, Sweden; Department of Cardio-Thoracic Surgery, Sahlgrenska University Hospital, Gothenburg, Sweden; Department of Clinical Sciences Lund, Skåne University Hospital, Lund University, Lund, Sweden; Department of Cardiothoracic Surgery, Skåne University Hospital, Lund University, Lund, Sweden; Department of Cardiothoracic and Vascular Surgery, Faculty of Medicine and Health, Örebro University Hospital, Örebro, Sweden; Department of Cardiothoracic Surgery, Karolinska University Hospital and Molecular Medicine and Surgery, Karolinska Institutet, Stockholm, Sweden

**Keywords:** Cox-maze IV, Atrial fibrillation, Stroke, Cerebral bleeding

## Abstract

**OBJECTIVES:**

Analysis of the long-term risks of ischaemic stroke and cerebral bleeding in patients with atrial fibrillation after mitral valve surgery and concomitant Cox-maze IV procedure.

**METHODS:**

In total, 397 patients with symptomatic degenerative mitral valve insuffciency and atrial fibrillation, underwent mitral valve surgery and Cox-maze IV in Sweden between 2009 and 2017. In this retrospective nationwide analysis, patients were followed in national patient registers until 30 September 2022.

**RESULTS:**

There were 4 deaths within 30 days (1.0%). Mean follow-up was 8.7 (0.1–13.4) years. Survival without ischaemic stroke or cerebral haemorrhage at 5 and 10 years were 90% and 74%, respectively. Nineteen patients experienced an ischaemic stroke, of which 4 were fatal. Five of 34 patients (14.7%) with a history of stroke preoperatively experienced ischaemic stroke during follow-up. The linearized rate of ischaemic stroke per patient-year was 0.6% and was similar regardless of left atrial appendage closure during surgery or whether a mechanical valve was inserted. The observed ischaemic stroke rate was lower than the predicted rate for all CHA_2_DS_2_-VASc score groups. Fourteen patients suffered cerebral bleeding, of which 3 were fatal. Patients who experienced cerebral bleeding were older and had higher mechanical valve implantation rate than those without cerebral bleeding.

**CONCLUSIONS:**

Surgery for mitral valve insufficiency and concomitant Cox-maze IV can be performed with low perioperative risk. There is a low continuing risk of stroke long-term postoperatively that correlates with a higher CHA_2_DS_2_-VASc score. Patients with preoperative stroke are at increased risk of postoperative stroke despite atrial fibrillation surgery.

## Graphical Abstract

**Figure:**
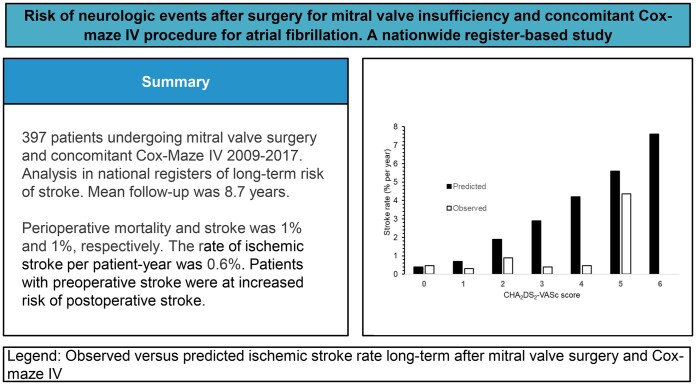

## INTRODUCTION

Atrial fibrillation (AF) is associated with an increased risk of stroke, heart failure, and mortality [1–3]. The Cox-maze III procedure, i.e. the cut-and-sew technique, to interrupt re-entrant wavelet circuits in the atria was developed during the 1990s and became the gold standard for surgical treatment of AF when unresponsive to medication therapy, electrical cardioversion or catheter ablation. Previous studies have documented that the Cox-maze III procedure effectively improves quality of life and reduces healthcare costs in patients with AF [4, 5]. Also, the ability of the Cox-maze III procedure to decrease the incidence of stroke is likely due to restoration of sinus rhythm and exclusion of the left atrial appendage (LAA) [6, 7]. During the past decades, the cut-and-sew technique evolved into the time-saving Cox-maze IV procedure, in which lesions in the atrial wall are created with cryo- or bipolar radiofrequency ablation. This simplification is especially useful if concomitant cardiac procedures are performed [8]. In patients with mitral valve disease and AF, concomitant Cox-maze IV ablation with mitral valve surgery has been recommended as a Class I indication in contemporary guidelines [9]. Recent register studies have shown a long-term survival benefit when performing surgical ablation for AF concomitant with other cardiac operations [10]; however, underlying reasons are partially unclear. The present study aimed to analyse the register-based long-term incidence of ischaemic stroke and cerebral bleeding after mitral valve surgery in patients with mitral valve insufficiency and preoperative AF who underwent concomitant Cox-maze IV procedure.

## MATERIALS AND METHODS

### Ethic statement

All data were anonymous before any analyses and obtained from the Swedish National Board of Health and Welfare on 2 November 2022. Informed consent was not required for analyses of register data. This study complied with the principles of the Declaration of Helsinki and was approved by the Regional Ethical Review Board in Stockholm (Dnr. 2018/608-31).

### Data source

Hospital records and operative notes were reviewed to correctly identify and include patients in the study group. All participants were followed up until death or through 30 September 2022, in the Swedish National Patient Registry (covering all hospitalizations in Sweden from 1972 and outpatient care from 1987) and the National Cause of Death Registry (all deaths in Swedish residents from 1961), respectively, by linking the unique personal identification number assigned to all individuals living in Sweden. Record linkages were performed by the Swedish National Board of Health and Welfare. The International Classification of Diseases (ICD-10) codes were used to identify prior and concurrent medical conditions. The Cause of Death Register was used to determine the cause and date of every death. The date of hospital admission for stroke or cerebral bleeding was used for the actuarial calculations. In cases of sudden-onset neurological symptoms, computed tomography of the brain was used to determine if the cause was ischaemic stroke or intracranial haemorrhage.

### Patients

This retrospective nationwide multicentre observational analysis included 397 patients with symptomatic degenerative mitral valve insufficiency and AF who underwent combined mitral valve surgery (repair or replacement) and a biatrial Cox-maze IV procedure, with or without concomitant tricuspid valve surgery (repair or replacement) in Sweden between 2009 and 2017. Patients with alternative lesions such as isolated pulmonary vein isolation or other concomitant cardiac procedures were excluded. Pre-, peri- and early postoperative data were collected and analysed by members of the Swedish Arrhythmia Surgery Group, and all 7 Swedish cardiothoracic centres performing Cox-maze IV procedure participated in the study.

### Surgical technique

The operations were performed via midline sternotomy and on cardiopulmonary bypass. The Cox-maze IV biatrial lesion pattern was performed as described previously [8, 9, 11]. The lesions in the atria were accomplished through either cryoablation (argon-based CardioblateVR CryoFlex Surgical Ablation Probe, Medtronic Inc., MN, USA, or nitrous oxide-based cryoablation probe, AtriCure Inc., Mason, OH, USA) (*n* = 360, 90.7%) or bipolar radiofrequency ablation (Synergy, AtriCure Inc., Mason, OH, USA) (*n* = 37, 9.3%). Closure of the LAA was frequently performed, according to surgeon preference, in some patients using a closing device (AtriClip Left Atrial Appendage Exclusion System; AtriCure, Inc. Mason, Ohio, USA). After discharge from combined mitral valve surgery and Cox-maze IV, the patients continued anticoagulation treatment with warfarin (68%), non-vitamin K antagonist oral anticoagulation (15%), aspirin (6%), or no anticoagulation in 11%.

### Definitions

Baseline patient characteristics were obtained from patient records. Follow-up registry information for acute myocardial infarction was defined by ICD-10 codes I21.0 to I22.9, hospitalization for heart failure by I50 to I50.9, cerebral bleeding by I60.0 to I62.9, ischaemic stroke by I63. Supplementary Material, Table S2, lists all ICD-10 codes used for the analyses. Body mass index was calculated as the weight in kilograms divided by the square of height in metres. The preoperative surgical risk was assessed using the European System for Cardiac Operative Risk Evaluation Score II (EuroSCORE II) [12]. The CHA_2_DS_2_-VASc [congestive heart failure, hypertension, age ≥75 years (doubled), diabetes mellitus, prior stroke or transient ischaemic attack or thromboembolism (doubled), vascular disease, age 65–74 years, sex category] score for risk of stroke was calculated from patient characteristics at the time of admission [13].

### Statistical methods

Data are presented as the arithmetic mean and standard deviations or for skewed distributions as the median and interquartile ranges. Student’s *t*-test or the Mann–Whitney *U*-test was used for continuous variables. The Yates-corrected chi-squared test for proportions was used to analyse categorical variables. The Kaplan−Meier product-limit method estimates were used to calculate cumulative survival with 95% confidence intervals, and the Cox-Mantel test was applied to differences [14]. The Fisher’s exact test was used to analyse differences between ablation techniques. Calculations were performed using STATISTICA 13 (Stat Soft, Dell, TX, USA).

## RESULTS

### Early outcomes

The baseline characteristics in relation to the CHA_2_DS_2_-VASc scores for the 397 patients who underwent surgery for mitral valve insufficiency and concomitant Cox-maze IV procedure are listed in Table 1. One-fourth of the patients were females, and 71% had non-paroxysmal AF. Previous catheter ablation with late recurrence was reported in 3.5% of patients. Thirty-four patients (8.6%) had a history of preoperative stroke or transient ischaemic attack. Based on gender, male patients had higher creatinine levels, better functional class but lower calculated EuroSCORE, and less frequent tricuspid valve insufficiency than female patients (Supplementary Material, Table S1). Mitral valve repair was performed in 86%, and a mechanical valve was implanted in 11% (Table 2). Concomitant tricuspid annuloplasty was performed in 44% of the patients. The LAA was closed in 203 patients (51.1%). A closing device was used in 70 patients (15.8%), and in the remaining cases, the LAA was sutured from inside the left atrium. The 30-day mortality was 1.0%, with 4 early deaths from myocardial infarction and heart failure. Perioperative complications were relatively few and included reoperation for bleeding 6%, stroke/transient ischaemic attack directly after the operation 1% and need for dialysis 2% (Table 2). Rhythm outcome at discharge was sinus/nodal rhythm in 70%, AF/atrial flutter in 17% and permanent pacemaker in 12% of patients.

**Table 1:** Baseline characteristics in 397 patients who had mitral valve surgery for mitral insufficiency and concomitant Cox-maze IV in relation to CHA_2_DS_2_-VASC score

Variable	Cox-maze IV		CHA_2_DS_2_-VASc score 0, 1		CHA_2_DS_2_-VASc score ≥ 2		*P-*Value
	(*n *=* *397)		(*n *=* *188)		(*n *=* *209)		
	Mean	SD	Mean	SD	Mean	SD	
Age (years)	65.7	9	60.6	8.0	70.2	7.3	<0.001
BMI (kg/m^2^)	25.5	4.1	25.3	4.2	25.7	4.0	0.34
Creatinine (µmol/l)	91.3	23	89.4	20.2	92.5	25.4	0.18
	Median	IQR	Median	IQR	Median	IQR	
Duration AF (months)	5	1–12	3	1–12	7	2–15	<0.001
EuroSCORE II	1.82	0.03–3.80	0.96	0.02–2.25	2.11	0.04–5.13	<0.001
	*n*	%	*n*	%	*n*	%	
Female gender	93	23.4	14	7.4	79	37.8	<0.001
Hypertension	123	31.0	16	8.5	107	51.2	<0.001
Diabetes mellitus	14	3.5	1	0.5	13	6.2	0.005
Tricuspid insufficiency	116	29.2	34	18.1	82	39.2	<0.001
AF^a^	302	76.1	137	72.9	165	78.9	0.19
Paroxysmal AF	115	29.0	61	32.4	54	25.8	0.18
Non-paroxysmal AF	282	71.0	127	67.6	155	74.2	0.18
Previous							
Stroke/TIA	34	8.6	3	1.6	31	14.8	<0.001
Myocardial infarction	10	2.5	2	1.1	8	3.8	0.15
Cardiac surgery	16	4.0	7	3.7	9	4.3	0.97
Catheter ablation	14	3.5	8	4.3	6	2.9	0.64
NYHA I	21	5.3	11	5.9	10	4.8	
NYHA II	144	36.3	85	45.2	59	28.2	
NYHA III	211	53.1	83	44.1	128	61.2	0.001
NYHA IV	6	1.5			6	2.9	
LVEF >50%	235	59.2	120	63.8	115	55.0	
LVEF 31-50%	137	34.5	51	27.1	86	41.1	0.01
LVEF 21-30%	5	1.3	3	1.6	2	1.0	

a Heart rhythm upon admittance.

AF: atrial fibrillation; BMI: body mass index; EuroSCORE: European System for Cardiac Operative Risk Evaluation Score; LVEF: left ventricular ejection fraction; NYHA: New York Heart Association; SD: standard deviation; TIA: transient ischaemic attack.

**Table 2:** Perioperative data in 397 patients who had mitral valve surgery for mitral insufficiency and Cox-maze IV during 2009–2017

Variable	Mean	SD	
Cardiopulmonary bypass (min)	169	50	
Aortic cross-clamp time (min)	119	36	
Valve procedure	*n*	**%**	
Mitral valve repair	340	86	
Mechanical mitral valve	45	11	
Bioprosthetic mitral valve	12	3	
Tricuspid valve repair	174	44	
Tricuspid valve replacement	4	1	
Early postoperative complications			
Early reoperation for bleeding	25	6	
Stroke	5	1	
Renal replacement therapy	7	2	
Mediastinitis	4	1	
Permanent pacemaker ≤30 days	44	11	

SD: standard deviation.

### Follow-up

**Figure 1:**
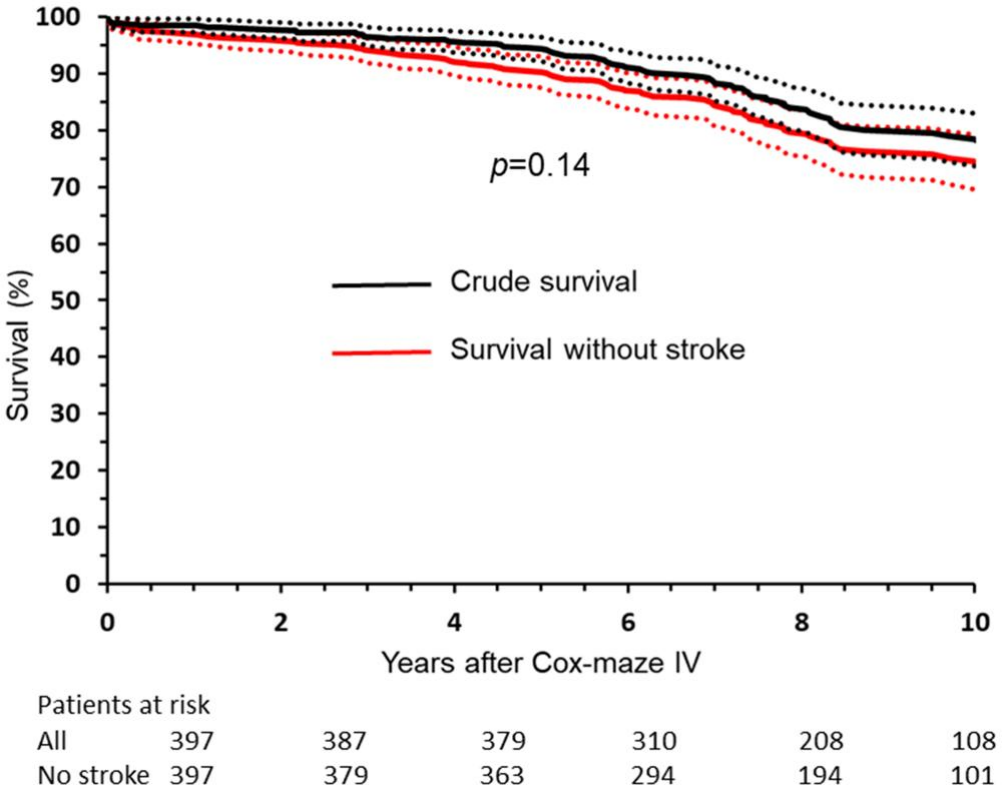
Crude survival and survival without ischaemic stroke after Cox-maze IV. The 95% confidence intervals are indicated.

The 393 early survivors were followed in patient registers for a mean of 8.3 (range 0.1–13.4) years. There were 78 late deaths: 42 from heart failure, 18 from malignancies, 10 from myocardial infarction, 3 from ischaemic stroke and 3 from cerebral bleeding. Overall survival at 5 and 10 years was 94% and 77%, respectively. Survival without ischaemic stroke or cerebral haemorrhage at 5 and 10 years was 90% and 74%, respectively (Fig. 1).

Nineteen patients suffered ischaemic strokes (4.8% of early survivors) during follow-up. One patient suffered an early stroke on postoperative day 8, and the remaining strokes occurred 4 months to 8 years after the operation (Supplementary Material, Fig. S1). Four of the ischaemic strokes were fatal (21.1%). Five of 34 patients (14.7%) with a history of stroke before the operation had an ischaemic stroke after the surgery, and 2 had cerebral bleeding (Table 3).

**Table 3:** Characteristics in patients with and without stroke or cerebral bleeding during follow-up

	No event (*n* = 364)		Stroke (*n* = 19)		*P*-value*	Cerebral bleeding (*n* = 14)		*P-*value*
	Mean	SD	Mean	SD		Mean	SD	
Age (years)	65.2	9.0	69.4	7.5	0.05	72.5	7.5	0.003
Body mass index	25.5	4.2	26.6	4.2	0.28	24.6	2.2	0.39
Creatinine (µmol/l)	91.0	22.8	97.7	18.1	0.21	90.4	25.6	0.92
EuroSCORE II	2.4	2.8	3.1	3.7	0.45	2.6	2.5	0.89
	*n*	%	*n*	%		*n*	%	
Paroxysmal AF	105	28.8	3	15.8	0.22	7	50.0	0.16
Non-paroxysmal AF	259	71.2	16	84.2	0.22	7	50.0	0.16
Female gender	89	24.5	2	10.5	0.27	2	14.3	0.58
Hypertension	111	31.0	5	26.3	0.90	7	50.0	0.21
Diabetes	12	3.3	1	5.3	0.85	1	7.1	0.98
Preop. stroke	27	7.4	5	26.3	0.01	2	14.3	0.27
Mechanical mitral valve	38	10.4	2	10.5	0.71	5	35.7	0.01
Left atrial appendage closed	182	50.0	11	57.9	0.66	10	71.4	0.19

* Comparison to those without an event.

AF: atrial fibrillation; EuroSCORE: European System for Cardiac Operative Risk Evaluation Score; SD: standard deviation.

Fourteen patients experienced cerebral bleeding (3.6% of early survivors) 2 months to 9 years after the operation (Supplementary Material, Fig. S2), 3 of which were fatal (20%). Patients who experienced cerebral bleeding were older and had a higher rate of mechanical valve implantations than those without cerebral bleeding after the operation. Almost half of the patients who experienced cerebral bleeding had hypertension.

The total follow-up time was 3272 years with a linearized rate of ischaemic stroke per patient-year of 0.6%, which did not differ significantly if the LAA was closed (0.6%) or not (0.5%) (*P *=* *0.86), or if a mechanical valve was used (0.5%) or not (0.6%) (*P *=* *0.79). Patients who did not have the LAA closed had a longer preoperative duration of AF, higher calculated EuroSCORE, more frequent paroxysmal AF, and longer cardiopulmonary bypass times than those in whom the appendage was closed (Table 4). There was no significant difference in ischaemic stroke rate between patients undergoing radiofrequency ablation and cryoablation (0/37 vs. 19/356, *P* = 0.15).

**Table 4:** Characteristics in 397 patients who had mitral valve surgery and Cox-maze IV in relation to closure of the left atrial appendage (LAA) during the operation

Variable	LAA closed (*n *=* *203)		LAA not closed (*n *=* *194)		*P*-value
	Mean	SD	Mean	SD	
Age (years)	66.0	9.1	65.3	8.9	0.49
Body mass index (kg/m^2^)	25.5	3.8	25.6	4.4	0.77
Creatinine (µmol/l)	91.8	25.3	90.7	19.6	0.62
Cardiopulmonary bypass (min)	162	52	174	48	0.02
Aortic cross-clamp time (min)	117	34	121	38	0.34
	Median	IQR	Median	IQR	
Duration AF (months)	3	1–11	7	1–15	0.002
EuroSCORE II	0.10	0.03–2.05	2.31	0.03–4.78	0.002
	*n*	%	*n*	%	
Female gender	49	24.1	44	22.7	0.53
Hypertension	73	36.0	50	25.8	0.09
Diabetes	10	4.9	4	2.1	0.32
Previous stroke/TIA	17	8.4	17	8.8	0.97
Paroxysmal fibrillation	75	36.9	40	20.6	0.001
Non-paroxysmal	128	63.1	154	79.4	0.001
Follow-up					
Ischaemic stroke	11	5.4	8	4.1	0.61
Cerebral bleeding	10	4.9	4	2.1	0.62

EuroSCORE: European System for Cardiac Operative Risk Evaluation Score; SD: standard deviation; TIA: transient ischaemic attack.

**Figure 2:**
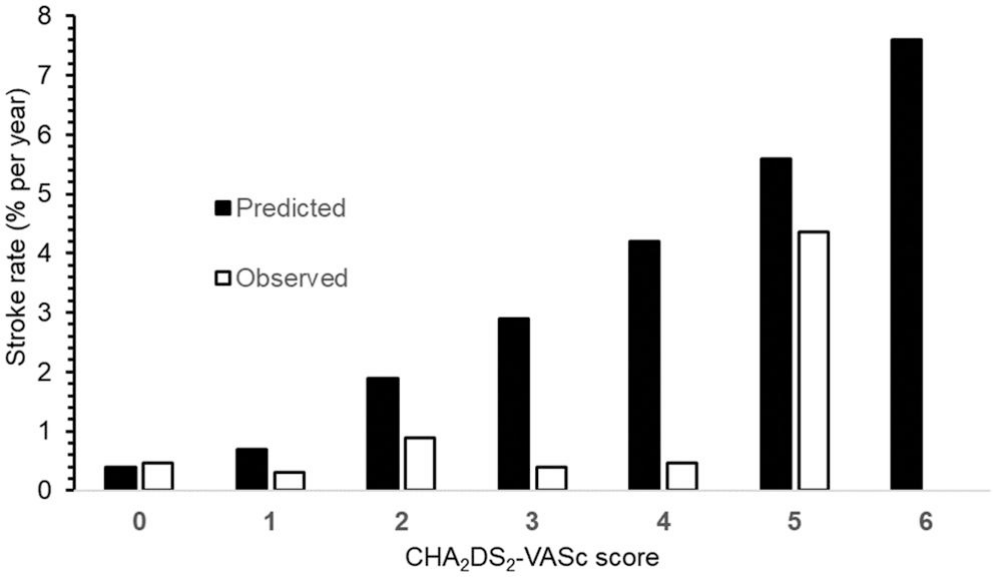
Observed versus predicted [15] stroke rate after mitral valve surgery and Cox-maze IV in 397 patients.

The number of ischaemic strokes and patients in each CHA_2_DS_2_-VASc score group are shown in Table 5. Only 7 patients had a CHA_2_DS_2_-VASc score 5, 2 of whom subsequently had strokes. None of the patients with a CHA_2_DS_2_-VASc score of 6 had a stroke. The observed stroke rate during follow-up stratified by the CHA_2_DS_2_-VASc score was lower than predicted in all score groups, compared with Swedish patients with AF without anticoagulant treatment [15] (Fig. 2).

**Table 5:** Linearized rates of ischaemic stroke in 397 patients

	CHA_2_DS_2_-VASC score	No. patients	No. events	Follow-up years	% Risk/patient-year
	0	77	3	647	0.5
	1	111	3	958	0.3
	2	108	8	895	0.9
	3	61	2	496	0.4
	4	28	1	212	0.5
	5	7	2	36	4.4
	6	5	0	28	
Total			19	3272	0.6

## DISCUSSION

This study covers the entire Swedish experience with mitral valve surgery for mitral insufficiency and concomitant Cox-maze IV procedures performed in 397 patients via median sternotomies during 8 years from 2009 to 2017 at 7 surgical centres. The quality of registration and validity of diagnoses in our national health registers are high and allow for virtually 100% coverage of the Swedish healthcare system. The validity of the diagnosis of stroke in the register has been evaluated to have a positive predictive value of 98.6% [16]. By linking personal identification number to national health registers, we obtained complete follow-up data until the date of death, and all survivors were followed up through September 2022. Every death or hospitalization after an operation for cerebral bleeding or stroke could be traced using ICD-10 codes.

In the present study, we found a low early mortality rate (1%) and perioperative stroke risk (1%) after mitral valve surgery and the Cox-maze IV procedure. Other authors have confirmed the safe use of routinely adding Cox-maze IV to other cardiac surgery [17]. Furthermore, in our study, the overall linearized yearly rates of stroke was 0.6%, in patients followed for almost 9 years postoperatively. Neurological events occurred during the entire follow-up period after the operation without any clusters of stroke or cerebral bleeding. As a comparison, Ad and colleagues reported 96.6% freedom from embolic stroke in 473 mitral valve and Cox-maze IV patients up to 7 years postoperatively (0.4% per patient-year) with many patients off anticoagulation [18].

Stratified by the CHA_2_DS_2_-VASc score, the observed rate of ischaemic stroke after mitral valve surgery and the Cox-maze IV procedure in our study was generally lower than predicted, compared to a previous report of 152 153 Swedish patients with AF who did not receive anticoagulant therapy followed for 5 years [15]. In that study, the risk of ischaemic stroke was 3.2% per patient-year, with increasing predicted risk related to higher CHA_2_DS_2_-VASc scores. The findings of lower observed ischaemic stroke rates than predicted are consistent with a previous follow-up by our group of Swedish patients undergoing stand-alone Cox-maze III surgery for AF [6]. Although the stroke rate was low in our present study cohort, patients with CHA_2_DS_2_-VASc scores of 5 had a high postoperative risk of stroke, but this subgroup was very small. Notably, one out of 6 patients who had experienced a stroke before the operation suffered an ischaemic stroke after the operation. These findings suggest that patients with preoperative stroke or higher CHA_2_DS_2_-VASc scores should be followed up more closely after surgery and possibly be anticoagulated more intensely. In addition to emboli originating in the heart and left atrium, stroke is strongly associated with factors such as old age, hypertension and peripheral vascular disease. Patients with a previous stroke are evidently at a high risk for subsequent stroke [13].

Whitlock *et al.* [19] demonstrated a lower risk of ischaemic stroke or systemic embolism with concomitant LAA occlusion performed during cardiac surgery than without it in a randomized trial including 4811 patients with AF and CHA_2_DS_2_-VASc scores ≥2 undergoing cardiac surgery followed for 4 years. Friedman *et al.* [20] reported that LAA occlusion, compared to no closure, was associated with a lower risk of readmission for thromboembolism over 3 years in 10 524 patients with a median age of 76 years who underwent cardiac surgery. Similarly, Mehaffey *et al.* [21] reported that surgical ablation and LAA closure were associated with lower 3-year mortality rates and readmission for stroke than after LAA closure alone in 103 382 patients over 65 years with AF undergoing cardiac surgery. In contrast to previous reports, we found a similar rate of ischaemic stroke regardless of whether the LAA was closed or not during surgery. In our cohort, the decision to close the LAA was based on surgeon preference, but it seemed that not performing LAA occlusion was more common in patients with higher operative risk and longer cardiopulmonary bypass times (possibly indicating more complex operations). The fact that many patients in this study did not have LAA occlusion is a limitation in interpreting overall stroke outcomes, given the proven significance of LAA occlusion in other contemporary studies. The lack of difference in stroke outcomes in our study between LAA occlusion or not may be related to the small sample size and relatively rare complications over time.

### Limitations

In this study evaluating neurologic events, we did not have valid information about long-term rhythm results or late freedom from AF. It is challenging to identify atrial arrhythmia recurrence late postoperatively after Cox-maze surgery, as symptoms and rhythm monitoring methods may vary. Instead, this retrospective study focused on national register analyses of mortality and neurological complications. The diagnoses of stroke were valid, but we did not know the cause of stroke in each patient, whether it was cardiac thrombi, peripheral vascular disease, or lacunar infarction. Also, we could not discern differences in stroke outcome between ablation methods used for the Cox-maze IV procedure, but the subgroups were very different in size and the study was not designed for such an analysis. Furthermore, most patients were discharged after surgery with anticoagulant treatment, but we had no information on compliance with prescribed medications and also lacked data on systemic embolization. It is conceivable that patients operated early in the series were treated mainly with warfarin and patients operated later were prescribed non-vitamin K antagonist oral anticoagulation, which could have had an impact on the rate of postoperative ischaemic stroke and cerebral bleeding. Patient risk factors, calculated CHA_2_DS_2_-VASc scores and risk of thromboembolic events may have changed during the years following the operation. Finally, these patients analysed in national registers are relatively uniform and the results may not extrapolate to other more heterogeneous patient populations.

## CONCLUSION

Surgery for mitral valve insufficiency and concomitant Cox-maze IV procedure can be performed with low perioperative risk. A low risk of stroke after surgery correlates with a high CHA_2_DS_2_-VASc score. Patients with preoperative stroke are at increased risk of postoperative stroke despite undergoing AF surgery.

## Supplementary Material

ivae189_Supplementary_Data

## Data Availability

The data underlying this article will be shared on reasonable request to the corresponding author.

## Notes

All authors are members of the Swedish Arrhythmia Surgery group.

## ABBREVIATIONS

AF: Atrial fibrillation
EuroSCORE: European System for Cardiac Operative Risk Evaluation Score II
ICD: International Classification of Diseases
LAA: Left atrial appendage
