# Supplementary material for: Risk of neurologic events after surgery for mitral valve insufficiency and concomitant Cox-maze IV procedure for atrial fibrillation. A nationwide register-based study
Source: Interdiscip Cardiovasc Thorac Surg. 2024 Nov 18;39(6):ivae189. doi: 10.1093/icvts/ivae189 (PMC11661525; doi:10.1093/icvts/ivae189)
Supplement: ivae189_Supplementary_Data [file ivae189_supplementary_data.docx]

**Supplementary Table S1.** Characteristics in men and women who had mitral valve surgery and Cox-maze IV during 2009 – 2017.

|  | *Men*  *(n=304)* | |  | *Women*  *(n=93)* | | *p Value* |
| --- | --- | --- | --- | --- | --- | --- |
|  | *Mean* | *SD* |  | *Mean* | *SD* |  |
| *Age (yrs)* | 65.1 | 8.9 |  | 67.5 | 9.0 | 0.02 |
| BMI (kg/m^2^) | 25.5 | 3.8 |  | 25.7 | 4.9 | 0.72 |
| Creatinine (µmol/L) | 93.7 | 22.6 |  | 82. | 22.9 | <0.001 |
|  | *Median* | *IQR* |  | *Median* | *IQR* |  |
| ^a^Duration AF (Mo) | 4 | 1-12 |  | 8 | 2-24 | 0.02 |
| Euroscore II | 1.59 | 0.03-3.10 |  | 2.71 | 0.06-5.6 | <0.001 |
|  | *n* | *%* |  | *n* | *%* |  |
| Hypertension | 86 | 28.3 |  | 37 | 39.8 | 0.05 |
| Diabetes mellitus | 11 | 3.6 |  | 3 | 3.2 | 0.89 |
| Tricuspid insufficiency | 80 | 26.3 |  | 36 | 38.7 | 0.03 |
| ^b^Atrial fibrillation | 231 | 76.0 |  | 71 | 76.3 | 0.95 |
| Paroxysmal fibrillation | 89 | 29.3 |  | 26 | 28.0 | 0.91 |
| Non-paroxysmal AF | 215 | 70.7 |  | 67 | 72.0 | 0.91 |
| Stroke /TIA | 24 | 7.9 |  | 10 | 10.8 | 0.52 |
| Myocardial infarction | 7 | 2.3 |  | 3 | 3.2 | 0.91 |
| NYHA I | 15 | 4.9 |  | 6 | 6.5 |  |
| NYHA II | 120 | 39.5 |  | 24 | 25.8 |  |
| NYHA III | 152 | 50.0 |  | 59 | 63.4 | 0.03 |
| NYHA IV | 4 | 1.3 |  | 3 | 2.6 |  |
| LVEF >50% | 180 | 59.2 |  | 55 | 59.1 |  |
| LVEF 31-50% | 102 | 35.6 |  | 35 | 37.6 | 0.55 |
| LVEF 21-30% | 5 | 1.6 |  |  |  |  |

^a^ Preoperative duration of atrial fibrillation
^b^ Heart rhythm on admittance
BMI: body mass index; AF: atrial fibrillation; TIA: transient ischemic attack: NYHA: New York Heart Association; LVEF: Left ventricular ejection fraction

| **Supplementary Table S2.** Definition of diagnoses/comorbid conditions. Primary and secondary diagnoses from the National Patient Register from 1987 and onward. | |
| --- | --- |
|  |  |
| **Myocardial infarction** |  |
| ICD 9 codes: | 410 |
| ICD 10 codes: | I21 to I21.9 |
|  |  |
| **Stroke** |  |
| ICD 9 codes: | 430 to 438 |
| ICD 10 codes: | I60 to I69.9 |
|  |  |
| **Heart failure** |  |
| ICD 9 codes: | 425, 428 |
| ICD 10 codes: | I50 to I50.9, I42 to I43.9, I25.5, K76.1, I11.0, I13.0, I13.2 |
|  |  |
| **Atrial fibrillation** |  |
| ICD 9 codes: | 427D |
| ICD 10 codes: | I48 to I48.9 |
|  |  |
| **Chronic obstructive pulmonary disease** |  |
| ICD 9 codes: | 490 to 496 |
| ICD 10 codes: | J44 to J44.9 |
|  |  |
| **Hypertension** |  |
| ICD 9 codes: | 401 to 405 |
| ICD 10 codes: | I10 to I15.9 |
|  |  |
| **Hyperlipidemia** |  |
| ICD 9 codes: | 272 |
| ICD 10 codes: | E78 to E78.9 |
|  |  |
| **Peripheral vascular disease** |  |
| ICD 9 codes: | 440 to 446 |
| ICD 10 codes: | I65 to I65.9, I71 to I71.9, I73.8, I73.9 |
|  |  |
| **Percutaneous coronary intervention** |  |
| ICD 9 codes: | 3080 |
| ICD 10 codes: | FNG00 to FNG06 |
|  |  |
|  |  |
| **Diabetes mellitus** |  |
| ICD 9 codes: | 250 |
| ICD 10 codes: | E10 to E14.9 |
|  |  |
| **Cancer** |  |
| ICD 9 codes: | 140 to 208 |
| ICD 10 codes: | C00 to C97.9 |
|  |  |
|  |  |
|  |  |

**Supplementary Figures and Figure legends**


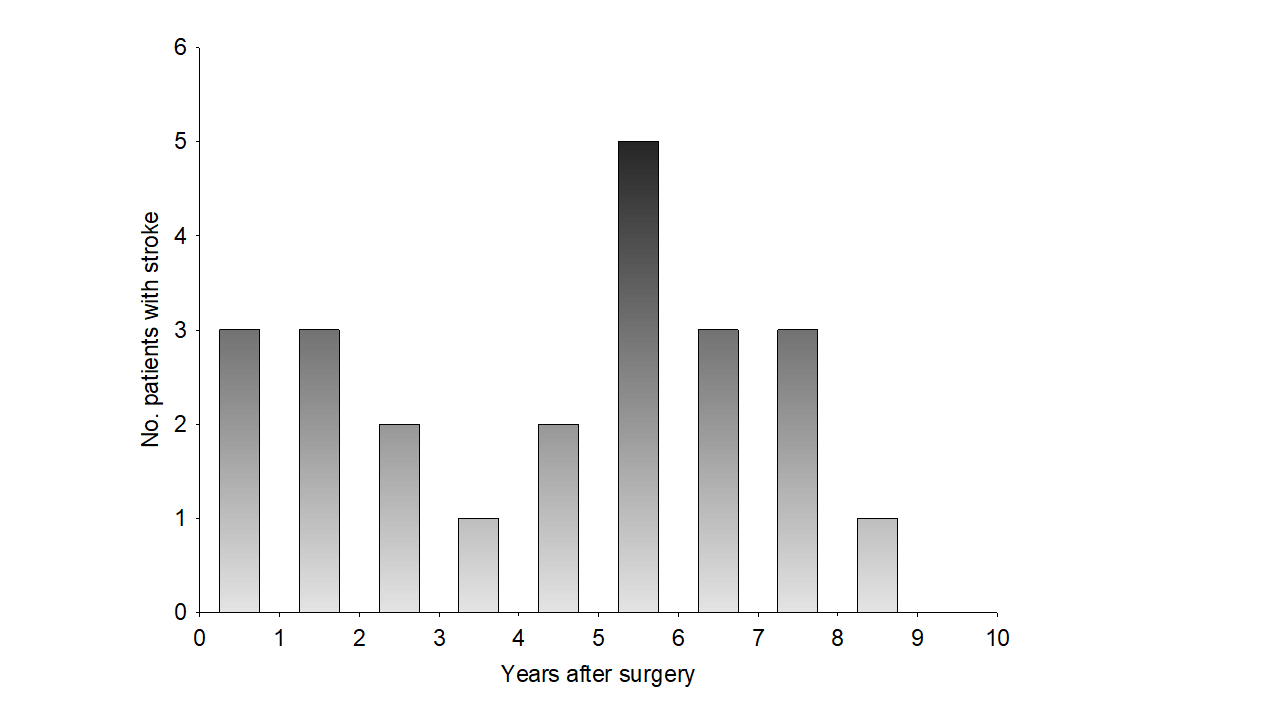


**Fig S1.** Year of stroke after mitral valve surgery and Cox-maze IV


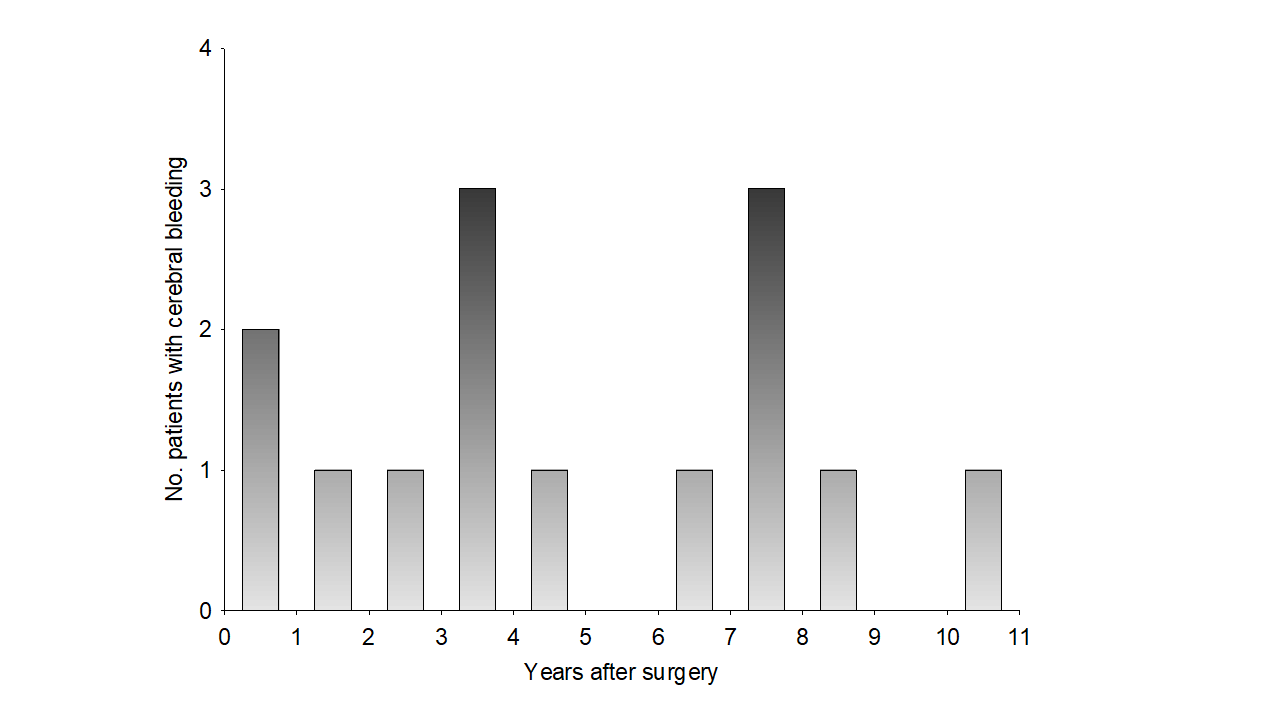


**Fig S2.** Year of cerebral bleeding after mitral valve surgery and Cox-maze IV
